# Appearance of levator ani muscle subdivision defects on level III vaginal support structures in women with and without pelvic organ prolapse: an MRI study

**DOI:** 10.1007/s00192-023-05533-1

**Published:** 2023-04-29

**Authors:** L. Horcicka, M. Krcmar, M. Nemec, L. Hympanova, J. Feyereisl, L. Krofta

**Affiliations:** 1 Third Faculty of Medicine, Charles University, Ruska 2411/87, 100 00 Prague, Czech Republic; 2 Institute for the Care of Mother and Child, Podolské nábřeží 157, 147 00, Prague, Czech Republic

**Keywords:** Level III, Levator ani muscle, Magnetic resonance imaging, Attachment patterns

## Abstract

**Introduction and hypothesis:**

Injury of the levator ani muscle (LAM) is a significant risk factor for pelvic organ prolapse (POP). The puborectalis (PRM) and pubovisceral (PVM) subdivisions are level III vaginal support structures. The null hypothesis was that there is no significant difference in patterns of LAM subdivisions in healthy nulliparous women. Secondarily, we evaluated the presence of different LAM injury in a POP-symptomatic cohort.

**Methods:**

This retrospective magnetic resonance imaging study included: 64 nulligravidae without any pelvic floor dysfunction (PFD) and 526 women of various parity with symptomatic POP. Primary outcome was PVM and PRM morphology on the axial planes: the attachment site on the pubic bone, and the visible separation/border between the PVM and PRM. The attachment was scored as “normal” or “abnormal”. The “abnormal” attachment was divided in two types: “type I”—loss of the muscle substance, but preservation of the overall muscle architecture—and “type II”—muscle detachment from the pubic bone.

**Results:**

The puboanal muscle (PAM) subdivision was evaluated as a representative part of the PVM. The PAM and PRM attachments and separation were distinguished in all asymptomatic nulliparae. PAM and PRM attachments did not significantly differ. POP group characteristics were parity 1.9 ± 0.8, instrumental delivery 5.6%, hysterectomy or POP surgery 60%, all Pelvic Organ Prolapse Quantification (POP-Q) stages, LAM defect 77.6% (PRM: 77.1%; PAM: 51.3%). Type I injuries were more frequent (PRM 54.7%; PAM 53.9%) compared with type II (PRM 29.4%; PAM 42.1%).

**Conclusions:**

A LAM defect was present in 77.6% of women with symptomatic POP. In PRM and PAM subdivisions type I injury was more frequent than type II.

## Introduction

The levator ani muscle (LAM) is a critical component of vaginal support structures [1]. Correctly identifying each part of the LAM is important in determining the effect of vaginal delivery-induced injuries to specific parts of the muscle. Kearney et al. in 2004 evaluated LAM anatomy described by various authors. They established a terminology consensus based on muscle origin and insertion [2]. The following three origin-insertion pairs are sufficient to describe the LAM divisions in women: the pubovisceral (PVM), puborectal (PRM) and iliococcygeal (ICM) muscles. The PVM has three subdivisions: puboperineal, pubovaginal and puboanal. The Lawson´s term pubovisceral eliminates the misleading name pubococcygeus and implies that the function of this muscle is to elevate the vagina and anorectum, not to move the coccyx [3]. Each LAM subdivision has a unique origin-insertion pair that determines its mechanical line of action [4]. The functional consequence of a LAM muscle injury depends on the region of muscle affected [5]. The PRM and PVM subdivisions are a part of level III vaginal support structures [6, 7]. They are attached to the periosteum of the pubic bone by fibrous enthesis. The detailed histology of the attachment demonstrates that tensional loading in a posteroinferior direction during the second stage of labour is predominant in those areas [8]. The tensile stretch leads to significant structural changes, including muscle tears or even avulsion. Histochemical analysis focused on the pubic origin of the LAM revealed two types of maternal LAM injuries. In “type I” injury some of the muscle mass is lost owing to muscle atrophy. “Type II” injury involves muscle detachment from the pubic bone due to excessive tension created during the second stage of delivery [9].

High-resolution magnetic resonance imaging (MRI) allows a detailed view of the LAM subdivisions in vivo. MRI studies have shown that birth-induced stretch injury causes characteristic changes in attachment patterns at the site where the muscle is directly attached to the pubic bone via a fibrous enthesis. The main goal of this study was to assess the attachment patterns of PRM and PVM in a characteristic injury zone on the inner surface of the pubic bone. We tested the null hypothesis that there is no significant difference in patterns of muscle LAM subdivisions in healthy nulliparous women. Secondarily, we evaluated the presence of different LAM injury in a symptomatic pelvic organ prolapse (POP) cohort.

## Materials and methods

### Study design, sample size, and inclusion and exclusion criteria

This study was approved by the institutional scientific and ethics committee (EK UPMD 3/2013 on 03.10.2013). Informed consent was obtained from all participants. A retrospective, observational, single-centre study was conducted between November 2014 and September 2022 at the Institute for the Care of Mother and Child in Prague.

The first group consists of 64 young gravida 0 para 0 women without any pelvic floor dysfunction (PFD) symptoms or a history of vaginal or perineal surgery, this group will be called asymptomatic nulliparae. The second group consists of 530 women, who were referred to the institution’s urogynaecological outpatient clinic with clinical symptoms of POP and underwent MRI examination of the pelvic floor during the study period. This group will be referred to as POP-symptomatic.

Exclusion criteria for both groups were previous abdominal or vaginal POP or stress urinary incontinence reconstructive surgery with synthetic mesh insertion, because these factors may distort pelvic organ support tissues at level III. Inclusion criteria for POP-symptomatic women were: vaginal prolapse symptoms, clinical POP signs, minimal interval of 6 months from last vaginal delivery, and minimal interval of 1 year from last POP reconstructive surgery.

### The MRI protocol

The MRI protocol was a high-resolution 3 T MRI scan (Phillips Achieva TX series), taken in the supine position. The imaging parameters were as follows: repetition time 5,331 ms, 375 phase encodes, 24-cm field of view, and 2-mm slice thickness, with no gap between slices. MRI sequences at rest were acquired in sagittal, coronal and axial planes. In general, axial images are the most suitable option for assessing the relationship between the genital tract and pelvic walls at each level, including the nature of the attachments [10]. The women had not received bowel or bladder preparation and had to urinate 30 min before the examination.

### Procedures, data analysis and outcome measures

Primary outcome was the analysis of LAM subdivision attachment points to the os pubis in asymptomatic nulliparae. Secondary outcome was the analysis of LAM subdivision defects in the POP-symptomatic group.

There were two parts to the study. The purpose of the first was to gain familiarity with the anatomy of the puboanal muscle (PAM) and PRM subdivisions. For these reasons we used 64 MRI scans of asymptomatic nulliparae. The measurements were performed for both PRM and PAM and all were measured in millimetres above the arcuate pubic ligament (APL). The APL is a reference structure for plane 0. Measurements started at plane 0. The distance was measured from the slide 0 to the first slide where the attachment appeared by movement to other axial slices of known thickness, without gaps. First, the location of the distal muscle attachment was measured. Second, the proximal attachment was measured and last the location of the central part of the attachment. Based on measures of distal and proximal attachments the attachment length was calculated. Separately, the visibility of the puborectal muscle loop was evaluated in a single scan; the loop was either visible in one slide or not.

**Fig. 1:**
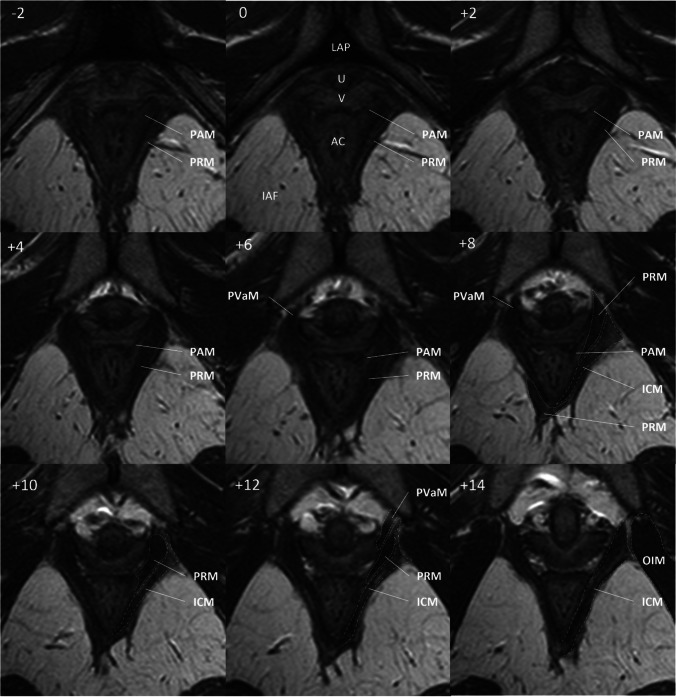
Axial scan of a subject (28 years) without pelvic floor dysfunction symptoms and no history of pregnancy. The scan planes are shown in the left upper corner relative to the arcuate pubic ligament (plane 0). The level of the scan plane in millimetres caudal to the ligament is indicated by a negative number. The notation + indicates the number of millimetres cranial to the plane 0. No scans are omitted between planes −2 and + 14. *U* urethra, *V* vagina, *AC* anal canal, *PAM* puboanal muscle, *PRM* puborectal muscle, *IAF* ischioanal fossa, *PVaM* pubovaginal muscle, *ICM* iliococcygeal muscle, *OIM* obturator internus muscle

In detail, the morphology of the PVM and PRM subdivisions were analysed in the most distally placed axial planes of the bony pelvis. A reference structure for the scan plane 0, the APL, had been identified. The notation + for other axial scan planes indicated the number of millimetres cephalad to the plane 0. The axial planes demonstrated a clear view of both subdivisions. The PAM PVM subdivision could be easily distinguished; therefore, we focused on this part of the PVM. The PAM and PRM subdivisions originate from the inner surface of the pubic bones where the PAM attachment points are localised medial to the PRM. The PAM subdivision goes medially to the PRM and passes into the intersphincteric space. The PAM can be seen in one slice. The PRM appears as a loop around the proximal part of the anal canal and the entire muscle mass is not always visible in one slice. The fibres between the PRM and PAM are not parallel, and the muscle subdivisions angles relative to the horizontal line range from 41° for the PVM to −19° for the PRM [5]. This fact represents a potential limitation for one plane imaging of both subdivisions simultaneously. Figure 1 shows an illustrative view of the PRM and PVM subdivisions in the axial planes. The morphology of the PRM and PAM was analysed for the presence of characteristic features. The following aspects were evaluated: differences in the attachment of the PAM and PRM subdivisions to the pubic bone, and a visible separation/border between muscle subdivisions.

**Fig. 2:**
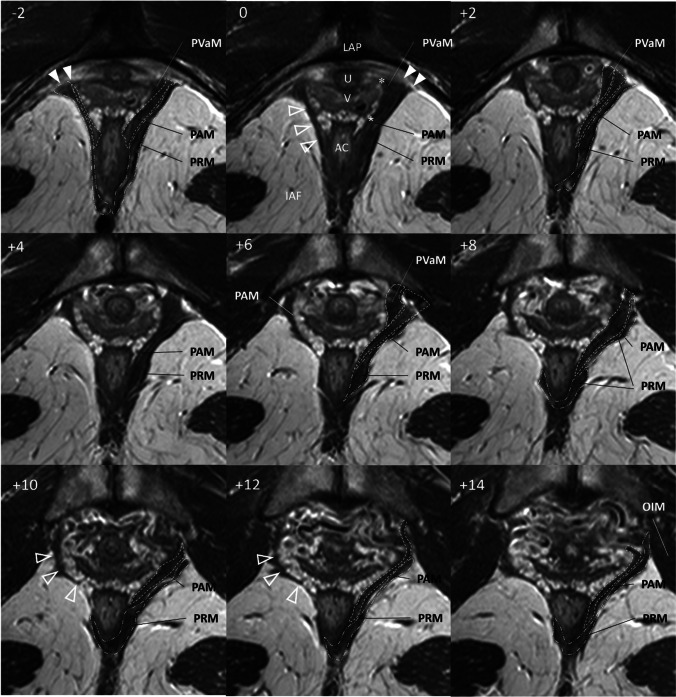
Exemplary of type I injury. Axial scan of subject (32 years) with pelvic organ prolapse with a history of two vaginal deliveries. Pelvic Organ Prolapse Quantification: Aa 1, Ba 1, C −2, D −1, Ap −1, Bp −1, gh 4, pb 3,5, TVL 12. The scan planes are shown in the left upper corner relative to the arcuate pubic ligament (plane 0). The level of scan plane in millimetres caudal to the ligament is indicated by a negative number. The notation + indicates the number of millimetres cranial to the plane 0. No scans are omitted between planes −2 and +14. The panels −2, 0 and + 2 show intact origin points of PAM and PRM bilaterally (*white triangle*). The intact attachment between the PVM and the vaginal wall is also visible. This place is indicated by *asterisks*. Thinning of the PAM is demonstrated by an *clear triangle*. The muscle bulk is missing, but the pelvic architecture is preserved. *LAP* arcuate pubic ligament, *U* urethra, *V* vagina, *AC* anal canal, *PAM* puboanal muscle, *PRM* puborectal muscle, *IAF* ischioanal fossa, *PVaM* pubovaginal muscle, *ICM* iliococcygeal muscle, *OIM* obturator internus muscle

**Fig. 3:**
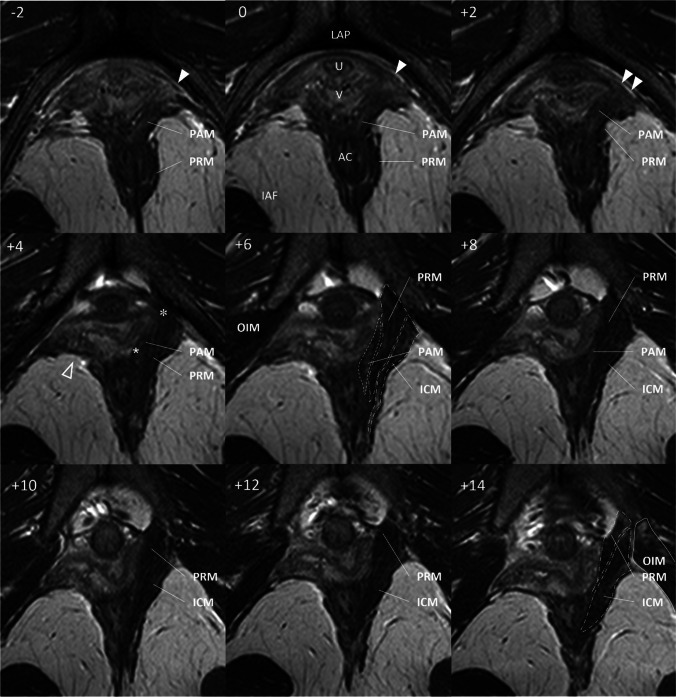
Example of a type II injury. The defect is shown on the right side. Axial scan of a subject (36 years) with pelvic organ prolapse with a history of one vaginal delivery. Pelvic Organ Prolapse Quantification: Aa 1.5, Ba 2, C −3, D −2, Ap −2, Bp −2, gh 4, pb 4, TVL 9. The scan planes are shown in the left upper corner relative to the arcuate pubic ligament (plane 0). Level of the scan plane in millimetres caudal to the ligament is indicated by a negative number. The notation + indicates the number of millimetres cranial to the plane 0. No scans are omitted between planes −2 and +14. The panels −2, 0, +2 show intact origin points of the puboanal muscle (*PAM*) and puborectal muscle (*PRM*) on the left side (*white*). The loss of this attachment for both subdivisions is shown on the right side. Panel +4: the missing muscle is denoted by the *clear triangle*. The intact attachment between the levator ani muscle (*LAM*) subdivisions and the vaginal wall is also visible. This place is indicated by *asterisks*. Panel +6: the intact LAM subdivisions are traced and labelled (PAM –––-, PRM –..–..–, iliococcygeal muscle [*ICM*] _ _ _ _ _ _). The normal pelvic architecture is damaged. The lateral right vaginal wall protrudes laterally and reach the OIM. *LAP* arcuate pubic ligament, *U* urethra, *V* vagina, *AC* anal canal, *IAF* ischioanal fossa, *PVM* pubovaginal muscle, *OIM* obturator internus muscle

In second part of the study, for each subject in the POP-symptomatic group PRM and PAM subdivision attachment in the axial scan plane were scored as “normal” or “abnormal”. The “abnormal” attachment patterns were selected according to gross architectural distortion of the muscle anatomy in two types: “type I”—loss of muscle substance and maintenance of the overall muscle architecture—and “type II”—muscle detachment from the pubic bone [9]. Both right and left LAM segments were evaluated separately. These LAM abnormalities are shown in Figs. 2 and 3.

All scans were evaluated independently by two researchers (M.K., L.H), who were blinded to a subject´s Pelvic Organ Prolapse Quantification (POP-Q) status. Final classification was only established when abnormal muscle morphology was found, as agreed upon by the two investigators. When the two examiners disagreed on the presence of an abnormality, re-examination of the scans were performed. Another examiner (L.K.), who was blinded to previous evaluations, viewed the questionable scans.

### Other examinations

The pelvic floor assessment was performed using the POP-Q system [11]. Knowing the shortcomings of the POP-Q staging system we divided the POP-symptomatic women according to vaginal wall placement in relation to hymenal ring. The Czech version of a validated questionnaire (the Pelvic Organ Prolapse Impact Questionnaire) was used to quantify POP symptoms. The pelvic examination during maximum straining excluded genitourinary prolapse in a group of asymptomatic nulliparae. Table 1 summarises the demographic, obstetrics and urogynaecological data of the analysed POP-symptomatic cohort (*N*=526). For descriptive purposes women were divided arbitrarily into age categories (Table 1).

**Table 1:** Characterisation of the POP-symptomatic group (*N*=526), including selected demographic, obstetric and urogynaecological details of pelvic organ prolapse (*POP*) reconstructive surgeries, Pelvic Organ Prolapse Quantification (*POP-Q*) staging POP-Q points. The POP-Q points are expressed in median and interquartile range (IQR). Prolapse group is additionally divided, based on clinical relevance into a group with prolapse above the hymenal ring and a group with prolapse at or below the hymenal ring

Parameter	POP symptomatic cohort
Demographic data	
Age (years)	57.1 (±13.1)
Age categories (years)	
20–30	14 (2.7%)
31–45	97 (18.4%)
46–55	80 (15.2%)
56–65	192 (36.5%)
66–75	113 (21.5%)
76–90	30 (5.7%)
Obstetrics details	
Parity	
Para 0	9 (1.7%)
Para I	123 (23.3%)
Para II	291 (55.2%)
Para III	89 (16.9%)
Para ≥IV	15 (2.8%)
Type of delivery	
No history of delivery	9 (1.7%)
Spontaneous	487 (92.4%)
Instrumental (forceps)	30 (5.7%)
Caesarean section	1 (0.2%)
Uterine status and previous POP reconstructive surgeries	
No surgery and uterus in situ	210 (39.8%)
1 Surgery and hysterectomy	177 (33.6%)
2 Surgeries and hysterectomy	106 (20.1%)
≥3 Surgeries and hysterectomy	19 (3.6%)
Surgeries without hysterectomy	15 (2.8%)
POP-Q staging	
Stage I	14 (2.7%)
Stage II	273 (51.9%)
Stage III	221 (42.0%)
Stage IV	18 (3.4%)
Lowest point of the prolapse	
Vaginal wall support	
Above hymen	21 (3.9%)
At or below hymen	505 (96.0%)
POP-Q points	
Aa	0 (−1.5 to 1.0)
Ba	0 (−1.5 to 1.5)
C	−3.0 (−5.0 to 0.0)
Ap	−1.0 (−2.0 to 0.0)
Bp	−1.0 (−2.0 to 1.0)
gh	5.0 (4.0—5.5)
pb	4.0 (3.5—4.0)
TVL	8.0 (7.0—9.0)
POP localisation - Compartment	
Only anterior	170 (32.3%)
Only central	11 (2.1%)
Only posterior	127 (24.1%)
Anterior and central	82 (15.6%)
Posterior and central	25 (4.8%)
Anterior and posterior	33 (6.3%)
All three compartments	78 (14.8%)

### Statistical analysis

All statistical analyses were performed using SPSS software (version 23.0; SPSS, Chicago, IL, USA). The symptomatic and asymptomatic groups were not compared, as demographic and urogynaecological parameters were by definition different between the selected study groups. For continuous, normally distributed variables in the asymptomatic nulliparous group, the paired samples test was used. Chi-squared test was used for analysis in the POP-symptomatic cohort to prove the independence in the contingency table with dichotomous variables.

A *p* value <0.05 was considered to be significant. Interobserver (L.H. and M.K.) agreement was obtained for attachment of the PVM and PRM subdivisions to the pubic bone, visible separation/border between muscle subdivisions, and type I/II attachment defect differentiation in a test/retest series in a blinded way on 50 women. The intraclass correlation coefficient (ICC) was used for this purpose.

## Results

### Basic population characteristics

The mean age and BMI of asymptomatic nulliparous women was 28.5 years (minimum 22, maximum 38, SD ±3.9) and 22.7 kg/m^2^ (minimum 17.8, maximum 38, SD ±3.2).

In women with POP symptoms the mean age was 57.1 years (minimum 27, maximum 88, SD ±13.1) and mean BMI 26.6 kg/m^2^ (minimum 17.3, maximum 41.7, SD ±4.2). Mean parity was 1.9 (SD ±0.8, range 0–6). Thirty (5.6%) subjects had an instrumental delivery. Only 39.8% of women had no history of previous hysterectomy or POP reconstructive surgery. POP-Q stages were: stage I, 2.7%, stage II, 51.9% and stage III and IV, 45.4%. The anterior compartment was most often affected. Sixty-nine percent of women had the anterior wall (alone or in combination with other compartments), 50% the posterior wall and 37.3% the central compartment involved in the prolapse. Detailed division is shown in Table 1.

### MRI outcomes: analysis of gravida 0 women without any PFD symptoms

Within the first part of the study, we were able to identify and distinguish the PAM and PRM subdivisions attachment to the pubic bone in all cases. We were also able to detect a visible separation between muscle subdivisions. No significant right-/left-side differences were found in any of these parameters. Table 2 summarises the specific features of normality for PAM and PRM subdivisions in asymptomatic nulliparous women. We did not find any statistically significant differences in the attachment of muscle subdivisions. Also, there were no type I or II injuries present.

**Table 2:** Measurements performed in the axial planes in the group of 64 gravida 0 young healthy women. Characteristic features of the levator ani muscle subdivisions, the puboanal (*PAM*) and the puborectal muscle (*PRM*) were assessed. The first three rows show measurements in millimetres above the arcuate pubic ligament, which is the reference structure for plane 0. Measurements were comparable in the PAM and PRM groups. Paired samples test was used for the comparison

Characteristic features	3 T MRI axial scan		
	Levator ani muscle subdivisions		*p*
	PAM	PRM	
Distal attachment placement (mm)	4.0 (±4.9)	4.8 (±4.7)	0.480
Proximal attachment placement (mm)	13.8 (±5.9)	14.9 (±5.3)	0.188
Location of the central part of the attachment (mm)	9.6 (±4.5)	9.7 (±4.4)	0.480
Attachment length in total (mm)	10.2 (±5.0)	9.4 (±3.9)	0.350
Puborectal muscle loop visible in single scan plane	–	54 (84.4%)	–

### MRI outcomes: analysis of POP-symptomatic cohort

In the second part of the study, during the study period, inclusion criteria were met by 530 POP-symptomatic women. Out of the 530 MRI scans, 4 could not be analysed because of signal artefacts.

The presence and type of LAM subdivision defects are shown in Table 3. In the POP-symptomatic cohort 77.6% (408 out of 526) LAM defects were detected. The proportion of cases with “abnormal” attachment patterns was significantly higher in the PRM than in the PAM subdivision (77.1 vs 51.3%).

**Table 3:** The assessment of defects of the levator ani muscle (*LAM*) and its subdivisions (level III vaginal support structures: puboanal muscle [*PAM*] and puborectal muscle [*PRM*]) in the POP-symptomatic cohort (*N*=526). The defects were evaluated at the origin-insertion points. The Chi-squared test was performed. The *p* value <0.05 indicates significance

	LAM	PRM		PAM		*p*
	*N* (%)	*N* (%)	95% CI	*N* (%)	95% CI	
No LAM defect	118 (22.4%)^a^					
LAM defect	408 (77.6%)					
No LAM defect		120 (22.8%)	19.2–26.4	256 (48.7%)	44.4–52.9	0.000
LAM defect		406 (77.1%)	73.6–80.8	270 (51.3%)	47.1–55.6	0.000
Type I injury						
Bilateral		175 (33.3%)	29.2–37.3	95 (18.1%)	14.8–21.3	0.000
Unilateral: right sided		39 (7.4%)	5.2–9.7	29 (5.5%)	3.6–7.5	0.000
Unilateral left sided		8 (1.5%)	0.5–2.6	6 (1.1%)	0.2–2.0	0.000
Type II injury						
Bilateral		81 (15.4%)	12.3–18.5	59 (11.2%)	8.5–13.9	0.000
Unilateral: right sided		23 (4.4%)	2.6–6.1	30 (5.7%)	3.7–7.7	0.000
Unilateral left sided		16 (3.0%)	1.6–4.5	14 (2.7%)	1.3–4.0	0.000
Combined (bilateral injury)						
Type I right + type II left		28 (5.3%)	3.4–7.2	17 (3.2%)	1.7–4.7	0.000
Type I left + type II right		36 (6.8%)	4.7–9.0	20 (3.8%)	2.2–5.4	0.000

^a^Not included two cases of PRM without and PAM with trauma

The most common type of abnormal PRM subdivision attachment patterns was bilateral type I. In 33.3% (175 out of 526) of POP-symptomatic women, bilateral loss of the muscle mass and maintenance of the overall muscle architecture were detected. In the PAM subdivision, bilateral type I abnormality was also the most common one, being found in 18.1% (95 out of 526) of the women. This type I pattern occurred simultaneously in both subdivisions in 14.6% (77 out of 526) cases. The second most common type of abnormal PRM subdivision attachment patterns was bilateral type II. In 15.4% (81 out of 526) POP-symptomatic women bilateral muscle detachment from the pubic bone was present. An identical situation regarding type II incidence was observed in the PAM subdivision. We found type II abnormal attachments in 11.2% of the cases (59 out 526). Type II pattern occurred simultaneously in both subdivisions in 10.5% of individuals (55 out of 526). In 138 women with unilateral or bilateral type I or type II abnormality of the PRM subdivision we detected 118 cases of an intact PAM subdivision. Only 2 cases (0.4%) of abnormal PAM attachment were associated with normal PRM subdivisions. A sub-analysis of the presence of LAM subdivision defects in women who had a vaginal wall below the hymenal ring is shown in Table 4. The difference in the incidence of abnormal attachment patterns was not statistically significant (Chi-squared test: PRM 0.066, PAM 0.156).

**Table 4:** The assessment of defects of the levator ani muscle subdivisions (level III vaginal support structures: puboanal muscle [*PAM*] and puborectal muscle [*PRM*]) in the POP-symptomatic cohort (*N*=526). The defects were evaluated at the origin-insertion points. Columns are divided in relation to two types of severity of prolapse. Low: includes the lowest point of the prolapse above the hymen; high: includes the lowest point at or below the hymen. The columns low and high for each muscle subdivision were compared using Chi-squared test; the results were not significant. *p* value <0.05 was significant

	PRM		PAM	
	Low POP stage (*n* = 21)	High POP stage (*n* = 505)	Low POP stage (*n* = 21)	High POP stage (*n* = 505)
No LAM defect	8 (38.1%)	112 (22.2%)	12 (57.1%)	244 (48.3%)
LAM defect	13 (61.9%)	112 (77.8%)	9 (42.9%)	261 (51.7%)
Type I injury				
Bilateral	2 (9.5%)	173 (34.3%)	1 (4.8%)	94 (18.6%)
Unilateral: right sided	1 (4.8%)	38 (7.5%)	0 (0.0%)	29 (5.7%)
Unilateral: left sided	0 (0.0%)	8 (1.6%)	1 (4.8%)	5 (1.0%)
Type II injury				
Bilateral	5 (23.8%)	76 (15.0%)	4 (19.0%)	55 (10.9%)
Unilateral: right sided	2 (9.5%)	21 (4.2%)	2 (9.5%)	28 (5.5%)
Unilateral: left sided	2 (9.5%)	14 (2.8%)	1 (4.8%)	13 (2.6%)
Combined bilateral type I and II injury				
Type I right + type II left	1 (4.8%)	27 (5.3%)	0 (0.0%)	17 (3.4%)
Type I left + type II right	0 (0.0%)	36 (7.1%)	0 (0.0%)	20 (4.0%)

We did not find a statistically significant difference in the incidence of abnormal attachment patterns between POP-symptomatic women of age categories 20–30 vs 31–45 years for either muscular subdivision (Chi-squared test: PRM 0.272; PAM 0.334). On the other hand, comparison of the age category 20–30 vs 46–55, 56–65, 66–75 and 76–90 has shown a statistically significant difference for both subdivisions (Chi-squared test: PRM subdivision 0.004, 0.001, 0.001 and <0.001; PAM subdivision 0.011, 0.006, 0.025 and 0.038).

We did not observe a statistically significant difference in the incidence of abnormal attachment patterns between POP-symptomatic women for both subdivisions with one versus two and one versus three or more deliveries (Chi-squared test: PRM subdivision 0.155 and 0.576; PAM subdivision 0.432 and 0.240).

The ICC comprised 20 POP-symptomatic and 30 asymptomatic nulliparous MRI examinations. The values were ranked between 0.85 and 0.96, with best agreement for attachment of the PVM and PRM subdivisions to the pubic bone in asymptomatic nulliparae. The lowest agreement concerned the evaluation of visible separation/border between the PRM and ICM muscle subdivisions.

## Discussion

This MRI study provides an insight into the normal and abnormal attachment patterns of LAM subdivisions on level III vaginal support. In 77.6% POP-symptomatic women the abnormal LAM subdivision attachment patterns on the pubic bone were present. The PRM subdivision was more often involved in the injury than the PAM component.

Assessment is possible because of the use of a highly standardised investigation technique in our settings. MRI description of the LAM has been published during the last 10–15 years [4, 5, 9, 10, 12]. The use of high-resolution 3 T MRI provides a wide range of signal intensity in regions of adjacent soft tissues, allowing excellent resolution. Slices measuring 2 mm thick and no gap between two slices reduced the probability that some structures would not be visible or be missing on the images.

Level III corresponds to the part of the distal vagina that extends 2–3 cm above the hymenal ring. At this level the vagina is fused to the medial surface of the LAM and compared with levels I and II, no intervening paracolpium is present [1]. The reason for this is the difference embryological derivation. This region arises from the urogenital sinus, whereas levels I and II are developed from Mullerian ducts [1]. Although for the sake of our study we have focused on level III alone, the three levels of vaginal support represent a continuum and are therefore interdependent.

A visible attachment of PRM and PAM subdivisions onto the pubic bone was detectable in 100% of women in the group of asymptomatic nulliparae. This is in contrast with the absence of a visible insertion in 10% of the 20 continent nulliparous women published by Tunn et al. [13]. Technical limitation of the 1.5 T with a slice thickness of 4 mm and a slice gap of 1 mm could explain the 10% and insertion <5 mm may be missed in some subjects. A visible separation of the PRM subdivision from the elements of the PAM was present in 100% of all asymptomatic nulliparae. The PRM subdivision always originates laterally to the PAM and passes laterally to the pubovisceral subdivision. The same observation was published by Margulies et al. [14]. The PRM fibre directions are oblique to the axial MRI scan plane; therefore, the entire PRM loop is not always visible in one slice. In 10 individuals (15.6%), the entire PRM loop was not visible in a single scan. The topography of PRM and PAM subdivision origins was analysed using a consistently visible reference structure, the APL. Related to the APL position, the origin sites of both muscle subdivisions do not differ. On average, both start and end in the same positions. Both origin sites are therefore at the same height as the pubis and the length of the attachment is also similar. There are differences in individual cases only. Our measurements are consistent with previous findings of Chou and DeLancey, showing that LAM normally attaches to the pubis from 0.5 to 2.0 cm above the APL [15].

The proportion of LAM defects on level III vaginal support structures was 77.6%. This observation is consistent with the work of DeLancey et al. They found that women with POP were more likely to have a LAM defect than controls. The incidence of LAM abnormalities was 70.9% [16]. However, their methodology differed from ours. In the mentioned work, 1.5 T MRI, slice thickness 4 mm, 1-mm gap between slices were used. They scored LAM defects using a system previously described for evaluating birth-associated damage in the axial plane [17]. The scoring of LAM defects was based on muscle mass reduction and the resulting grade is the sum of the scores of the right and left sides. The scoring system did not work with the abnormal attachment patterns that we used. However, the total numbers of normal and abnormal findings are comparable with our findings when we used our methodology. We have documented in our previous work, using DeLancey´s LAM defect scoring methodology, the incidence of LAM defects at level III in POP-symptomatic para I women of 67.1% for major and 12.8% for minor LAM trauma [18].

In relation to LAM components, in older publications the pubovisceral component included all muscles arising from the pubic bone, also the PRM. Our study follows the trend and clearly distinct PVM and PRM as already published [2]. There are two reasons why we evaluate the PRM subdivision separately. First, we respect the terminology consensus based on muscle origin and insertion published by Kearney et al. [2]. Second, the justification for considering PRM separately from the PVM component is because it is inserted differently. Each origin-insertion LAM subdivision pair has a specific mechanical function. Knowing which subdivision is damaged is necessary in order to understand how the injury might result in a specific type of PFD. This is the point where information regarding abnormal attachment patterns could be useful. This multivariate analysis will be part of our future study. The importance of level III structures has to do more with the ability of the LAM to keep the vagina closed than with the vagina´s ability to remain attached to its surrounding structures.

In “type I” injury some substance of the muscular subdivision can be locally damaged, which in turn leads to muscle atrophy due to denervation. The muscle is lost but the levator arch and vaginal shape at the level of the injury remains intact. “Type II” injury involves muscular detachment from the pubic bone and would involve a loss of the normal architecture of the pelvic sidewall. This important change in the supportive tissue represents an additional risk factor for POP development in these women. Identification and understanding of abnormal attachment patterns could be important for subsequent adequate therapeutic management strategies including surgical interventions in women with PFD. In type I abnormal attachment patterns where the muscular shape remains intact, physiotherapy can be beneficial. On the contrary, in type II injury, which includes a loss of critical attachment, early and intensive exercise therapy could exacerbate the separation between the muscle and its origin point. This hypothesis needs to be verified.

We found abnormal attachment patterns to be more common in the PRM than in the PAM subdivision. This seems logical as the stretch ratio during vaginal delivery in PRM is higher than in the PAM subdivision. The different subdivisions stretch ratios could explain the fact that within the groups 44.0% bilateral type I and 67.9% bilateral type II abnormal attachment were found in both subdivisions. This fact demonstrated that the muscular defect is not automatically transferred to all LAM subdivisions at level III and that they are involved individually. In the case of normal attachment patterns of the PRM subdivision in POP-symptomatic women, there is a very low chance (0.4%) of abnormal finding in the PAM subdivision. On the contrary, normal attachment patterns of the PAM subdivision may be accompanied by abnormal attachment patterns of the PRM subdivision (53.9%).

Type II abnormal attachment patterns at level III would involve a loss of the normal appearance of the vaginal shape. At this level, the vagina is fused with the structures that surround it. Unlike in the upper levels, there is no intervening connective tissue of the paracolpium that separates the vaginal wall from adjacent structures and the wall fuses with the medial margin of the LAM. Fully expressed type II abnormal attachment patterns of the PVM carries the vagina with it and is responsible for the lateral spill of the vagina [19]. We believe that this phenomenon does not occur in the case of a combination of type II abnormal attachment of one LAM subdivision with type I or normal patterns of another division on the same side. To develop this abnormal vaginal shape within level II, a paravaginal defect must be present at the same time. This hypothesis needs to be verified.

Regarding the frequency of PRM subdivision abnormalities, our work has different conclusions compared with already published work [20]. In published article defects in the PRM were not seen on MRI in women with major levator ani defects and POP. The explanation lies in differences in interpreting and labelling of the muscle. What we mark here as the ICM could be based on what other authors consider to be the PRM and what we mark as the PRM could be considered by others to be the PVM.

There were eight nulliparae in our POP-symptomatic group. In seven cases, hysterectomies have been performed in the past. In all seven cases, the central compartment was involved in the defect. In this group, PRM and PAM subdivisions showed normal attachment patterns.

The subjects were included in the prolapse group based on their clinical symptoms. Based on population-based studies of asymptomatic women it is only women with a vaginal wall at or below the hymenal ring who have a true prolapse [21]. Therefore, sub-analysis of groups with prolapse at or below the hymenal ring and above the hymenal ring was also performed (Table 4). The difference in the occurrence of abnormal attachment patterns was not statistically significant. Berger et al. showed that LAM defect grading is an important POP risk factor. They suggest that higher degrees of levator ani defects correlate most strongly with POP [22]. Within this study we cannot make straightforward conclusions on whether some type of injury is more or less frequent in relation to degree of POP, age or parity, as the appropriate control groups were not included, and we did not intend to investigate this. Analysis with division related to the hymenal ring was performed (Table 4).

The difference we found in abnormal attachment patterns between age categories is due to a higher proportion of type I attachment patterns in the older age categories. The reduced muscle thickness could also be a part of the aging process. Aging is a strong risk factor for POP and is associated with a reduction of skeletal muscle fibres and muscle strength [23]. The LAM is also a skeletal muscle. In all pelvic floor muscles signs of aging were detected, as well as a decrease in the predicted force production and fibrosis [24]. Indicated signs of skeletal muscle fibres could be also responsible for resting LAM bowl volume enlargement by 80% in older women [25]. This type of morphological and the resulting functional changes suggest that aging can decrease the resting tone of the pelvic floor muscles.

Parity is a key risk factor for POP. We did not find a statistically significant difference in the incidence of abnormal attachment patterns between POP-symptomatic women for both subdivisions with one or two and one or three and more vaginal deliveries. Our data suggest that the first vaginal birth might have the most significant effect on the condition of the muscles.

Lesser degrees of injury (mainly type I) do not occur more frequently in women with prolapse than in age- and parity-matched parous women. It is only greater degrees of injury are more common in women with prolapse than in those without [22]. Therefore, the important injuries are the type II injuries; thus women with lesser degrees of injury are not worried that they may cause problems. We suggest that aging might represent a risk factor.

The strengths of the study are primarily the large cohort of women with PFD. A cohort consisting of only ethnic Caucasian women represents the local population very well. Furthermore, a highly detailed and standardised 3 Tesla MRI protocol was used.

The methodology of the study has several limitations. This was not a population-based study; thus, the findings cannot be used to estimate the prevalence of muscular defects in the general population. The investigators were not blinded to the LAM subdivision status at the time of evaluation as it is not technically possible to deflect the LAM structure. This can represent a bias. Only the axial images were used for LAM assessment. The group was inhomogeneous in terms of POP defect localisation and the number of POP reconstructive surgeries. Subanalysis according to POP-Q parameters and surgery interventions is not part of the present work. Another limitation of our study is that the parous control group without prolapse was not included. In such a group type I and type II injury have been described [22].

The present study has provided a detailed morphological description of LAM attachment patterns on level III vaginal support structures in women with POP. General MRI description of the LAM has been published; however, not always in appropriate detail, which has now been achieved in the current 3 T MRI study.

In conclusion, under normal conditions 3 T MRI allows visualisation of PRM and PAM subdivisions on level III vaginal support structures. Both origin sites are at the same height on the pubis. In POP-symptomatic women on level III the morphology of PRM and PAM subdivisions shows two types of abnormal attachment patterns, which are less significantly pronounced in the PAM subdivision. Those types of changes were not observed in asymptomatic nulliparae. Owing to the limitations of this study, generalisation of the results should be made with care.

## Data Availability

Data available on request.

## Abbreviations

APL: Arcuate pubic ligament
ICC: Intraclass correlation coefficient
ICM: Iliococcygeal muscle
LAM: Levator ani muscle
MRI: Magnetic resonance imaging
OIM: Obturator internus muscle
PAM: Puboanal muscle
PFD: Pelvic floor dysfunction
POP: Pelvic organ prolapse
POP-Q: Pelvic Organ Prolapse Quantification
PRM: Puborectal muscle
PVM: Pubovisceral muscle complex
